# Clinical changes of TMD and condyle stability after two jaw surgery with and without preceding TMD treatments in class III patients

**DOI:** 10.1186/s40902-015-0008-2

**Published:** 2015-02-21

**Authors:** Sang-Yong Yoon, Jae-Min Song, Yong-Deok Kim, In-Kyo Chung, Sang-Hun Shin, Pusan Korea Pusan National University

**Affiliations:** Department of Oral and Maxillofacial Surgery, School of Dentistry, Pusan National University, 49 Busandaehak-ro, Mulgeum-eup, Yangsan, 626-870 Korea

**Keywords:** Orthognathic surgery, 2 jaw surgery, TMJ, Condylar stability, TMD

## Abstract

**Background:**

This study are to identify the symptomatic changes and condylar stability after 2 jaw surgery without preceding treatments for Temporomandibular joints(TMJ) in class III patients with the TMJ symptoms; and to assess therapeutic effect of 2 jaw surgery and the necessity of preceding treatment for alleviation of TMJ symptoms.

**Methods:**

30 prognathic patients with preexisting TMJ symptoms were divided into 2 groups according to presence or absence of preceding treatments before the surgery. We evaluated symptomatic changes on both TMJ by questionnaires and clinical examinations. And we reconstructed 3D cone beam computed tomography images before 2 jaw surgery, immediately after the surgery, and 6 months or more after the surgery with SimPlant software, and analyzed the stability of condylar position on 3D reconstruction model. Significances were assessed by the Wilcoxon signed rank test on SPSS ver. 20.0.

**Results:**

Both groups had favorable changes of TMJ symptoms after orthognathic surgery. And postoperative position of condyle had good stability during follow-up period.

**Conclusion:**

2 jaw surgery without preceding treatments for TMD can have therapeutic effect for TMD patients with class III malocclusion.

## Background

Many patients with dentofacial deformity often have various symptoms and signs on temporomandibular joints (TMJ) and its related structures. The symptoms and signs of temporomandibular disorders (TMD) typically include: (1) Pain during resting, palpation or joint movement, (2) TMJ noise such as clicking, popping, and crepitus, (3) Joint dysfunction such as limitation of mouth opening (LOM), jaw locking, and jaw deviations. TMD can be the manifestation of multifactorial dysfunction in oral and maxillofacial area [1], and the occlusion accounts for only a small portion. McNamara et al. [2] expected that the contribution of occlusal factors to TMJ symptoms is only 10 ~ 20%.

**Figure 1:**
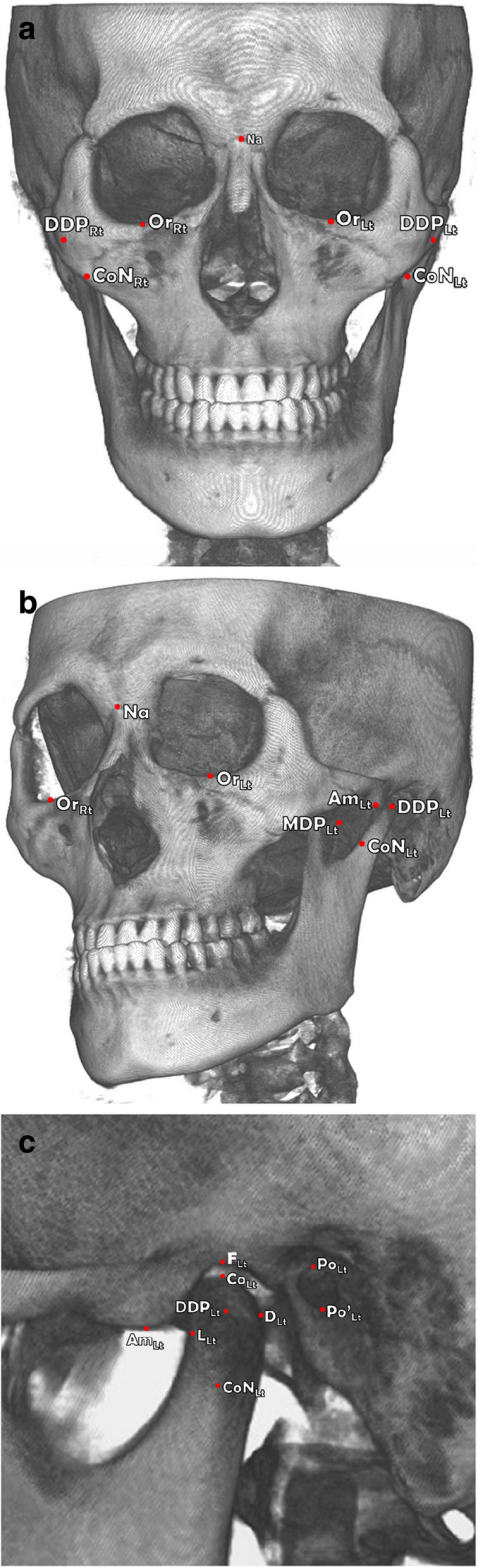
**Reference points on 3D reconstruction model.** **a**. Coronal view; **b**. Oblique view (Left); **c**. Sagittal view (Left).

**Figure 2:**
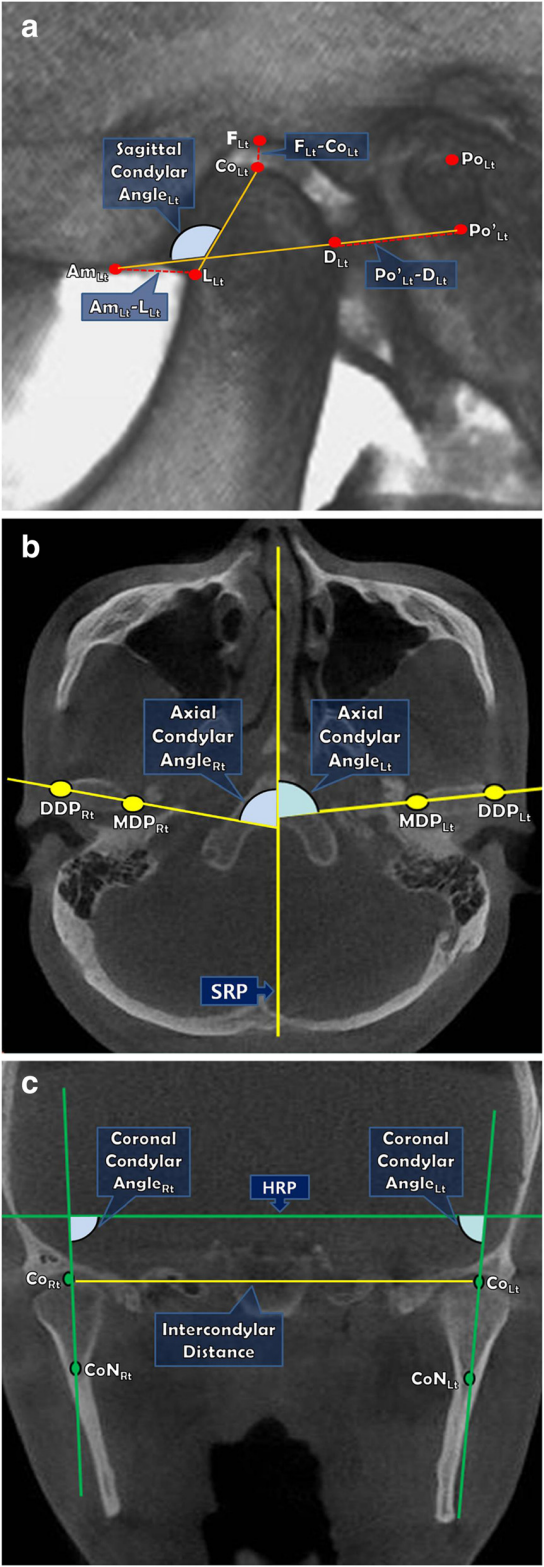
**Measurements on 3D CBCT images.** **a**. Sagittal view (Left); **b**. Axial view; **c**. Coronal view.

Orthognathic surgery, especially 2-jaw surgery is a treatment to resolve severe skeletal discrepancies through surgical corrections of maxillomandibular complex, and can change the congenital interocclusal relationship and condylar position. Many studies have been reported various degree of improvement, deterioration or no effect in symptoms and signs of TMD after orthognathic surgery [3-13]. Recently, it is generalized that there is no contraindication related with TMD in orthognathic surgery except for acute symptoms or inflammatory diseases on TMJ. But there is still controversial about the necessity of preceding treatment for stabilization of preoperative TMJ condition.

The purpose of this retrospective study are (1) to identify changes of clinical symptoms and signs and postoperative stability of condyle according to presence or absence of preceding treatments in 2-jaw surgery patients with prior TMD, and (2) to evaluate the necessity of preceding treatment for alleviation of TMD.

## Methods

### Subjects

The initial subjects consisted of 54 patients (18 males and 36 females) who complained of TMD before 2-jaw surgery. Inclusion criteria were: 2-jaw surgery by Le Fort I osteotomy and bilateral sagittal split ramus osteotomy(BSSRO), no history of orofacial trauma, check-up with cone beam computed tomography (CBCT) imaging at 3 periods (T0: preoperative period, T1: postoperative 1 week or less, T2: postoperative 6 months or more). Exclusion criteria were: congenital developmental disorders such as cranio-facial syndromes and clefting, inflammatory TMJ disease such as acute capsulitis and osteoarthritis. On the basis of the criteria, 32 patients were recruited finally. The patients were divided into two groups according to presence or absence of preceding treatments for the purposes of alleviation of TMD and stabilization of condyles before the surgery: The study group consisted of 15 patients that had no preoperative TMD treatments (sex: 4 males and 11 females, mean age: 24.8 ± 2.76 years, range: 21 - 31 years). And the control group consisted of 15 patients had been treated until the symptoms and signs of TMD alleviated (sex: 7 males and 8 females, mean age: 24.4 ± 4.29 years, range: 18 - 31 years). The treatments for stabilization of preoperative TMJ condition included medication therapy, physical therapy, splint therapy, and self-regulation therapy. This study was exempted by the Institutional Review Board at Pusan National University Dental Hospital, and we followed the guidelines of Helsinki Declaration in this study.

### Surgical procedure

All patients underwent 2-jaw surgery by 1 experienced surgeon from January, 2007 to June, 2012 in the clinic of Oral and Maxillofacial surgery, Pusan National University Dental Hospital. During the BSSRO, the mandibular proximal segments were manually repositioned and fixated with single miniplate (4 holes) & four monocortical screws (2.0 mm diameter) through intraoral approach. Intermaxillary fixation with the occlusion guided wafer was applied for 1 week after the surgery. Since the 1 week, the postoperative physical trainings for mandibular function were progressed gradually.

### Clinical examination of TMD

In this study, we collected the data by self-reported questionnaires and clinical and functional examinations: (1) TMJ pain during function(mouth opening or mastication), (2) TMJ noise on jaw movement, (3) LOM under 35 mm. The study group were examined in three times: before the preceding treatments for TMD, before surgery and 6 months after surgery. The control group were examined in two times: before surgery and 6 months after surgery. Self-reported questionnaire consisted of several questions regarding the subjective changes of TMJ symptoms. Clinical and functional examinations were performed for the diagnosis of TMD according to the Research Diagnostic Criteria for TMD (RDC/TMD) Axis I [14]. The severity of TMJ pain and noise was rated on Numerical Analogue Scales (NAS) composed of 11 rating points. The NAS ranged from 0 to 10 with 0 indicating ‘no symptom and sign’, 10 representing ‘worst possible symptom and sign’, and 5 documenting an intermediate level at ‘moderate symptom and sign’. The change of LOM was evaluated as three grades: ‘improvement (+)’, ‘deterioration (-)’, and ‘no change (0)’.

### Analysis of condylar position with 3D CBCT

The patients underwent 3D CBCT imaging with the closed mouth (Pax-Zenith 3D, VATECH, Korea). For ascertainment of positional changes in both condyles, dental CBCT (DCT) images were reconstructed with 3D dental image software (SimPlant Pro Crystal for Intel X86 Platform V13. 0. 1. 4, Belgium). On the basis of three reference planes and twenty-five reference points set up on 3D reconstruction model, fifteen measurements were obtained (Figures 1 and 2, Tables 1 and 2).

### Statistical methods

The data were analyzed with a commercial statistical software package (SPSS for windows ver. 20.0). Significances of differences between the times were assessed by the Wilcoxon signed rank test. The significant level is set at *P* < 0.05.

## Results

### Changes of clinical TMJ symptoms

Changes between preoperative and postoperative TMJ symptoms in 30 orthognathic patients are summarized (Tables 3, 4, 5 and 6). The significances of symptomatic changes after orthognathic surgery are analyzed statistically, but the statistical process of LOM was excluded because LOM was rare in patients (Table 7).

**Table 1:** **Definitions of reference points & planes**

**Reference planes**	**Description**
A. Reference planes	
Horizontal Reference Plane (HRP)	The plane constructed by Po_Rt._ - Or_Rt._ - Po_Lt._ (Frankfort plane)
Sagittal Reference Plane (SRP)	The plane perpendicular to FH plane & passing through Na – Ba line (Midsagittal plane)
Coronal Reference Plane (CRP)	The plane perpendicular to FH plane and Midsagittal plane passing through Na (Na - perpendicular plane)
B. Reference points	
Na	The most anterior point of nasofrontal suture on sagittal plane
S	The midpoint of the fossa hypophysealis
Ba	The midpoint on the anterior border of the foramen magnum
F_Rt._/F_Lt._	The most superior point of the (right/left) glenoid fossa
Co_Rt._/Co_Lt._	The most superior point of the (right/left) condyle
L_Rt._/L_Lt._	The most posterior point on anterior surface of (right/left) condyle
Po_Rt._/Po_Lt._	The most superior point of the (right/left) external auditory meatus
Po’_Rt._/Po’_Lt._	The most inferior point of the (right/left) external auditory meatus
Am_Rt._/Am_Lt._	The most inferior point of (right/left) articular eminence
MDP_Rt._/MDP_Lt._	The most medial point of (right/left) disc pole
DDP_Rt._/DDP_Lt._	The most distal point of (right/left) disc pole
CoN_Rt._/CoN_Lt._	The mid-point of (right/left) condylar neck on coronal view
Or_Rt._/Or_Lt._	The most inferior point of the (right/left) infraorbital margin
D_Rt._/D_Lt._	The intersecting point on posterior surface of condyle and Po - Am line (Right/Left)

**Table 2:** **Definitions of measurements**

**Plane**	**Measurement**	**Description**
Sagittal	Po’_Rt._ - D_Rt._/Po’_Lt._ - D_Lt._	Distance between Po’ & D
	F_Rt._ - Co_Rt._/F_Lt._ - Co_Lt._	Distance between F & Co
	Am_Rt._ - L_Rt._/Am_Lt._ - L_Lt._	Distance between Am & L
	Po’_Rt._ - Am_Rt._/Po’_Lt._ - Am_Lt._	Distance between Po’ & Am
	Sagittal condylar angle_Rt./Lt._	Angle composed of Co - L line & Po’ - Am line
Axial	Axial condylar angle_Rt./Lt._	Angle composed of MDP - DDP line & SRP
	Intercondylar Distance	Distance between Co_Rt._ & Co_Lt._
Coronal	Coronal condylar angle_Rt./Lt._	Angle composed of CoN - Co line & HRP

**Table 3:** **Clinical data in both TMJs, A. Distributions**

	**Study group**		**Control group**	
**Symptoms**	**T0**	**T2**	**T0**	**T2**
TMJ pain only	0 (0)	0 (0)	3 (20)	3 (20)
TMJ noise only	10 (66.7)	6 (40)	4 (26.7)	2 (13.3)
LOM only	0 (0)	0 (0)	0 (0)	0 (0)
TMJ pain and noise only	5 (33.3)	2 (13.3)	6 (40)	5 (33.3)
TMJ pain, noise and LOM	0 (0)	0 (0)	2 (13.3)	0 (0)
No symptoms	0 (0)	7 (46.7)	0 (0)	5 (33.3)

Numbers of patients (%).

**Table 4:** **Clinical data in both TMJs, B. TMJ pain and noise**

**B-1. Change of NAS values**				
	**Study group**		**Control group**	
**Symptoms**	**T0**	**T2**	**T0**	**T2**
TMJ pain	1.5 (0-7)	0.4 (0-4)	2.3 (0-7)	1.1 (0-4)
TMJ noise	4.0 (2-8)	1.2 (0-4)	3.1 (0-5)	0.9 (0-5)

Average (range).

**Table 5:** **Clinical data in both TMJs, B-2: Symptomatic change of both TMJs**

**Symptoms**	**Change**	**Study group**	**Control group**
	Improved	6 (20)	12 (40)
TMJ Pain	Deteriorated	0 (0)	4 (13)
	No change	24 (80)	14 (47)
	Improved	19 (63.5)	17 (56.5)
TMJ Noise	Deteriorated	0 (0)	1 (3.5)
	No change	11 (36.5)	12 (40)

Numbers of TMJs (%).

**Table 6:** **Clinical data in both TMJs, C LOM**

**Symptom**	**Change**	**Study group**	**Control group**
LOM	Improved	0 (0)	2 (13)
	Deteriorated	0 (0)	0 (0)
	No change	15 (100)	13 (87)

Numbers of patients (%).

**Table 7:** **Significance in changes of TMJ symptoms**

**Symptoms**	**Study group**			**Control group**		
	**T1-T0**	**T2-T0**	**T2-T1**	**T1-T0**	**T2-T0**	**T2-T1**
Pain	.034*	.034*	1.000	.241	.183	.842
Noise	.000*	.000*	.705	.000*	.001*	.886

*Significant difference by Wilcoxon signed rank test (*P* < 0.05).

#### Study group (patients without preoperative treatments for TMJ symptoms)

Ten patients (66.7%) experienced TMJ noises without TMJ pain before orthognathic surgery, and there were decrease to 6 patients (40%) after the surgery. 5 patients (33.3%) reported both TMJ pain and noise before the surgery were reduced to 2 patients (13.3%) after the surgery and those with no symptom increased from none to 7 (46.7%). The average scores of TMJ pain and noise were 1.5 (range : 0-7) and 4.0 (range : 2-8) on T0. But the scores were decreased to 0.4 (range : 0-4) and 1.2 (range : 0-4) on T2. All TMJs in study group had varying degrees of symptomatic alleviations and no deteriorations in comparison between the T0 and T2. The symptomatic decreases between preoperative and postoperative pain and noise (T0-T1, T0-T2) were significant statistically (*P* < .05).

#### Control group (patients with preoperative treatments for TMJ symptoms)

The number of patients with TMJ symptoms was decreased slightly and the number of patients with no TMJ symptoms was increased from none to 5 (33.3%) in control group. The average pain score was 2.3 (range : 0-7) on T0, and 1.1 (range : 0-4) on T2. The average noise score was reduced from 3.1 (range : 0-5) to 0.9 (range : 0-5) after 2 jaw surgery (T2). Although very few of the patients (2 of 11 patients with TMJ pain and 1 of 12 patients with TMJ noise) reported slight worsening comparing T0 versus T2, the majority of the patients were relieved or unchanged. Moreover two patients with preoperative LOM showed improvement unexceptionally. In the results of statistical analysis, changes of TMJ noise between T0 and T2 were statistically significant (*P* < .05) even though TMJ pain had no significant changes (*p* > .05).

**Table 8:** **Significances in changes of condylar positions**

**Measurement**	**Study group**			**Control group**		
	**T1-T0**	**T2-T1**	**T2-T0**	**T1-T0**	**T2-T1**	**T2-T0**
Po’_Lt._ - D_Lt._	.334	.594	.233	.057	.094	.100
Po’_Rt._ - D_Rt._	.589	.589	.865	.140	.211	.776
F_Lt._ - Co_Lt._	.008*	.036*	.307	.043*	.011*	.363
F_Rt._ - Co_Rt._	.001*	.009*	.058	.002*	.036*	.147
Am_Lt._ - L_Lt._	.670	.156	.156	.820	.495	.394
Am_Rt._ - L_Rt._	.001*	.233	.055	.281	.427	.053
Po’_Lt._ - Am_Lt._	.733	.256	.100	.551	.394	.460
Po’_Rt._ - Am_Rt._	.112	.910	.191	.910	.589	.820
Sagittal condylar angle (Lt.)	.256	.331	.798	.067	.256	.609
Sagittal condylar angle (Rt.)	.078	.910	.057	.733	.307	.211
Axial axis angle (Lt.)	.191	.865	.100	.191	.609	.100
Axial axis angle (Rt.)	.035*	.910	.052	.233	.650	.233
Intercondylar distance	.011*	.032*	.334	.061	.140	.100
Coronal axis angle (Lt.)	.712	.363	.427	.609	.281	.334
Coronal axis angle (Rt.)	.100	.650	.425	.363	.443	.156

*Significant difference by Wilcoxon signed rank test (*P* < 0.05).

### Changes of condylar position with analysis of 3d cbct

#### Surgical change (T0 – T1)

Between T0 and T1, study group had significant changes of F_Rt._ - Co_Rt._, F_Lt._ - Co_Lt._, Am_Rt._ - L_Rt._, Axial Axis angle (Rt.), Intercondylar Distance, and control group had significant changes of F_Rt._ - Co_Rt.,_ F_Lt._ - Co_Lt.,_. But, other measurements were not changed significantly during 2 jaw surgery.

#### Postoperative stability (T1 – T2)

Between T1 and T2, most measurements were not changed significantly after the surgery. Changes of only three measurements (F_Rt._ - Co_Rt._, F_Lt._ - Co_Lt._, Intercondylar Distance) were significant in both groups. Intercondylar distance significantly increased immediately after the surgery, but returned near the existing position during follow-up interval.

#### Definitive change of condylar position (T0 – T2)

The final changes of condylar positions were evaluated from identification of positional changes between T0 and T2. Any measurements of both groups did not have significant changes in this period (Table 8).

## Discussions

Many studies have suggested that surgical corrections of dento-facial deformities can improve the symptoms relating to TMJ pain and dysfunction [3-11]. However, Henrikson et al. [15] suggested that short-term decrease of the painful tenderness may be due to altered activity of the muscles, and Onizawa et al. [16] also speculated that alteration of TMJ sounds after orthognathic surgery were associated with postoperative reduction of mandibular mobility. Unlike these studies, we think that improvements of the TMJ symptoms are not solely due to postoperative reduction of muscular function or jaw mobility and may be relevant to the improvements of occlusal, skeletal and neuromuscular balance after the surgery. The aim of this study is to identify postoperative changes of TMD symptoms and condylar stability and to evaluate additional therapeutic effect of the surgery and necessity of preoperative TMD treatments on the orthognathic patients with TMD definitively.

The symptomatic results of this study are almost consistent with previous studies in which TMJ symptoms improved. Ueki et al. [17] found that the incidence of TMJ symptoms decreased after SSRO although SSRO did not change the disk position. According to Togashi et al. [18], the incidence of TMJ signs and symptoms significantly decreased from 29.5% before orthognathic surgery to 12.1% at one year after the surgery. Also, TMJ signs and symptoms decreased in 82.4% of symptomatic patients before the surgery. In this study, both symptomatic patients with and without preoperative TMD treatment had favorable changes of TMJ symptoms. Especially, TMJ noise decreased significantly in both groups. Although deteriorations of the symptoms were unusually shown, TMJ pain generally improved after the surgery. There were significant improvements and no deteriorations in study group, but a few deteriorations and unsignificant changes in control group. We speculated that it is the reason that the control group included some TMD patients with facial asymmetry. The comparison of LOM was little meaningful because of insufficiency of subjects with preexisting LOM.

Condylar repositioning during BSSRO can influence the changes of TMJ symptoms and positional stability of condyle during postoperative follow-up. Individual physiological adaptation also can affect positional changes of condyles during follow-up period. However, according to Nakata et al. [19], the physiological adaptation to the surgically corrected structures needs long time over two years. Because the follow-up time of this study was less than a year, we eliminated a factor of the physiologic adaptation. In this study, the condylar position of both groups during postoperative follow-up interval had good stability (*P* > .05) except for the distances between glenoid fossa and condyle (F – Co) in both sides. It seems that whether or not TMD treatments precede 2 jaw surgery in patients with prior TMD did not have any significant influences on postoperative stability of condylar position. We estimated that the significant changes of both F - Co measurements were because all patients in both groups underwent CBCT wearing the occlusal splint at T1 although showed significant changes (*P* < .05). In other words, we considered them as temporary increases due to wearing of the occlusal splint for preventing skeletal relapse during 1 month or less after the surgery. Intercondylar distance in study group significantly increased immediately after the surgery, but returned near the existing position during follow-up interval. Kim et al. [20] reported that condyle in the glenoid fossa had tendency to return to normal position during postoperative period in orthognathic patients. We used a miniplate and 4 monocortical screws for semirigid fixation so that functional stability and slight flexibility for enhanced long term TMJ function could be achieved [21].

2 jaw surgery can have favorable effects on TMJ symptoms in patients with dentofacial deformities. The results in this study showed that even if TMJ symptoms were not treated before the surgery, 2 jaw surgery could have therapeutic effects for TMJ symptoms while also provide good stability of condyles. This study has some differences from other similar studies. Firstly, we divided orthognathic patients into 2 groups contingent upon presence or absence of preoperative treatment for TMJ symptoms, unlike other studies in which TMJ symptoms concerned as the standard of classification. Secondly, we simultaneously analyzed postoperative stability of condylar position with 3D CBCT while examining changes of TMJ symptoms. On the other hand, there are also some limitations: (1) The number of the orthognathic patients without any preceding treatments for TMJ symptoms before the surgery were not enough. (2) We had only mandibular prognathic patients with class III malocclusion among various dentofacial deformities. (3) We did not use validated scales such as the modified Helkimo index, craniomandibular index, and the Research Diagnostic Criteria [14,22,23]. We collected only the symptomatic data by simple self-report form.

## Conclusions

This study showed improvements of preexisting TMJ symptoms and good condylar stability during the postoperative follow-up even though the patients were not treated for the TMJ symptoms before the surgery. Thus, We think that 2 jaw surgery patients with preexisting TMJ symptoms can have therapeutic effect exclusively attributed to the surgery unless the existence of acute and severe TMD before the surgery. However, the symptoms cannot be always improved and there would be some risk of symptomatic deterioration though the risk is very quite low.

## Abbreviations

TMJ: temporomandibular joints
TMD: temporomandibular disorders
LOM: limitation of mouth opening
BSSRO: bilateral sagittal split ramus osteotomy
CBCT: cone beam computed tomography
NAS: Numerical Analogue Scales
DCT: dental CBCT
